# Use of antimicrobial growth promoters in food animals and Enterococcus faecium resistance to therapeutic antimicrobial drugs in Europe.

**DOI:** 10.3201/eid0503.990303

**Published:** 1999

**Authors:** H C Wegener, F M Aarestrup, L B Jensen, A M Hammerum, F Bager

**Affiliations:** Danish Veterinary Laboratory, Copenhagen. hcw@svs.dk

## Abstract

Supplementing animal feed with antimicrobial agents to enhance growth has been common practice for more than 30 years and is estimated to constitute more than half the total antimicrobial use worldwide. The potential public health consequences of this use have been debated; however, until recently, clear evidence of a health risk was not available. Accumulating evidence now indicates that the use of the glycopeptide avoparcin as a growth promoter has created in food animals a major reservoir of Enterococcus faecium, which contains the high level glycopeptide resistance determinant vanA, located on the Tn1546 transposon. Furthermore, glycopeptide-resistant strains, as well as resistance determinants, can be transmitted from animals to humans. Two antimicrobial classes expected to provide the future therapeutic options for treatment of infections with vancomycin-resistant enterococci have analogues among the growth promoters, and a huge animal reservoir of resistant E. faecium has already been created, posing a new public health problem.


### Use of Antimicrobial Growth Promoters in Food Animals and *Enterococcus faecium* Resistance to Therapeutic Antimicrobial Drugs in Europe

**Henrik C. Wegener, Frank M. Aarestrup, Lars Bogø Jensen, Anette M. Hammerum, and Flemming Bager**
Danish Veterinary Laboratory, Copenhagen, Denmark

Supplementing animal feed with antimicrobial agents to enhance growth has been common practice for more than 30 years and is estimated to constitute more than half the total antimicrobial use worldwide. The potential public health consequences of this use have been debated; however, until recently, clear evidence of a health risk was not available. Accumulating evidence now indicates that the use of the glycopeptide avoparcin as a growth promoter has created in food animals a major reservoir of *Enterococcus faecium*, which contains the high level glycopeptide resistance determinant *vanA*, located on the Tn*1546* transposon. Furthermore, glycopeptide-resistant strains, as well as resistance determinants, can be transmitted from animals to humans. Two antimicrobial classes expected to provide the future therapeutic options for treatment of infections with vancomycin-resistant enterococci have analogues among the growth promoters, and a huge animal reservoir of resistant *E. faecium* has already been created, posing a new public health problem.

### Vancomycin-Resistant *Enterococcus faecium* (VRE)

In addition to being a member of the normal gut flora of nearly all warm-blooded animals (including humans), *E. faecium* has the ability to cause a wide range of infections, primarily serious infections in hospital patients (particularly in intensive care units). Increasing incidence of *E. faecium* infections has been associated with use of third-generation cephalosporins in hospitals (1). Enterococci are resistant to many antibiotics. In an increasing number of cases, vancomycin is the only treatment drug that remains effective. Because *E. faecium* was untreatable with practically all other antibiotics, the emergence of the first high level vancomycin-resistant *E. faecium* was of particular concern (2). By 1997, more than 15% of nosocomial enterococcal infections in U.S. hospitals were due to VRE (3).

Address for correspondence: Henrik C. Wegener, Danish Zoonosis Centre, Danish Veterinary Laboratory, Bülowsvej 27, DK-1790 Copenhagen V, Denmark; fax: +45-35-300-120; e-mail: hcw@svs.dk.

The vancomycin-resistant strain of *E. faecium* (VRE) contains the VanA gene cluster located at a mobile genetic element, a transposon designated Tn*1546*. Although other mechanisms and determinants of glycopeptide resistance have been found in *E. faecium*, in this article, VRE will refer to strains containing the *vanA* gene and Tn*1546*. First isolated in France in 1986, VRE were subsequently found in the United States in 1989, where they rapidly became a frequent cause of hospital infections (3). Like many other nosocomial pathogens, VRE were believed to originate and be maintained in hospitals and to have little, if any, association with the community. Nosocomial outbreaks and clonal spread in the United States supported this assumption (4). In Europe, however, even though serious incidents have occurred in some countries, VRE-associated hospital infections have not increased at the same rate and to the same proportion as in the United States (5). The first indication that the epidemiology of VRE may differ from that of other gram-positive organisms capable of causing hospital infections (e.g., methicillin-resistant *Staphylococcus aureus*)

came from the United Kingdom, where Bates et al. (6) reported isolating VRE from pig herds as well as from the farm environment. These scientists suggested that a community source of VRE may exist. Soon afterwards, Klare et al. (7) in Germany showed that VRE could be cultured frequently from pigs, poultry, and humans in the community and suggested that VRE may be associated with the use of glycopeptides as growth promoters in food animals.

### Antimicrobial Growth Promoters

Antimicrobial growth promoters (AGPs) are antibiotics added to the feed of food animals to enhance their growth rate and production performance. The mechanism by which AGPs work is not clear. AGPs reduce normal intestinal flora (which compete with the host for nutrients) and harmful gut bacteria (which may reduce performance by causing subclinical disease). The effect on growth may be due to a combination of both fewer normal intestinal flora and fewer harmful bacteria. The class of antimicrobial drugs used and the animal species involved may determine the relative importance of each mechanism (8). The quantity used in feed varies with each antimicrobial agent. In the European Union (EU), avoparcin 20 mg/kg and 40 mg/kg was approved for different age groups of pigs and chickens; the concentration is often referred to as "subtherapeutic" (not to be confused with sub-MIC levels). The resulting concentration in the gastrointestinal tract of the animal is sufficient to inhibit the susceptible bacteria and markedly affect the composition of the bacterial gut flora (8).

In Denmark, as well as in other countries, only a few glycopeptides have been used; for humans vancomycin and (to a lesser extent) teicoplanin have been used, and for animals avoparcin has been used exclusively as a feed additive for growth promotion. Avoparcin has not been used in animals in Sweden since 1986 because of a national prohibition of AGPs; in the United States, avoparcin was never approved because of its carcinogenic effects (9).

Few countries have accurate data on the use of antibiotics in animals and humans. In Denmark, 24 kg of active vancomycin was used for human therapy in 1994. In comparison, 24,000 kg of active avoparcin was used as a feed additive for animals (10). In Austria, an average of 582 kg of vancomycin was imported for medical purposes and 62,642 kg of avoparcin for animal husbandry per year from 1992 to 1996 (11). Thus, although there are more food animals than humans, the selective pressure favoring VRE in Europe can be estimated to be much higher in food animals than in humans. Data on the yearly use of vancomycin in the United States and in major European countries were recently published by Kirst et al. (12). Denmark, a small country, used more glycopeptide growth promotant (avoparcin) than all of Europe and the United States used for treating ill humans (vancomycin). Difference in denominators implies huge difference in use (10).

### Use of Avoparcin as a Growth Promoter and the Occurrence of VRE in Food Animals

The high selective pressure by the use of glycopeptides as growth promoters could explain the presence of VRE in food animals. We have conducted a number of studies to investigate the association between the use of avoparcin as a growth promoter and the occurrence of VRE.

In one study, eight poultry flocks raised conventionally and six raised without growth promoters were compared (13). No VRE was found in birds raised without growth promoters, whereas five out of eight conventional flocks contained VRE. Isolation rates in positive flocks were as follows: of five fecal samples tested, one to four (20%–80%) were positive. Twenty-two pig herds and 24 poultry flocks, half of which had used avoparcin and half of which had not, were compared by occurrence of VRE in fecal samples collected from animals of the herds and flocks. A strong and statistically highly significant association between the presence of VRE and the use of avoparcin was observed (14). Of 12 pig herds using feed with avoparcin, 8 had VRE, while of 10 herds not using avoparcin, 2 had VRE ($p = 0.043$, risk ratio [RR] 3.3; 95% confidence interval [CI]: 1.1, 10.0). In broiler farms where avoparcin was used, VRE was isolated from 11 of 12 fecal samples. In farms where avoparcin was not used, VRE was isolated in 2 of 12 samples ($p < 0.0006$; RR 5.5; 95% CI: 2.2, 13.9).

The association observed at the flock and herd level has also been observed at the country level. In countries where avoparcin had been used as a growth promoter, VRE could frequently be cultured from food animals, whereas in countries where avoparcin had not been used, VRE were not detected (Table 1). These findings are consistent with the hypothesis that use of avoparcin has created a reservoir for VRE in food animals.

Table 1. Avoparcin as a growth promoter in countries where the occurrence of vancomycin-resistant enterococci (VRE) in animal husbandry has been investigated

| Country | Avoparcin used | VRE in animal husbandry | Ref. |
|---|---|---|---|
| Belgium | + | + | 15 |
| Denmark | + | + | 13,14 |
| Finland | + | + | 16 |
| France | + | + | 17 |
| Germany | + | + | 7 |
| Great Britain | + | + | 6 |
| The Netherlands | + | + | 18 |
| Norway | + | + | 19 |
| Sweden | - | - | 20 |
| United States | - | - | 21,22 |

### Transmission of VRE from Animals to Humans

Can an animal reservoir in itself be regarded a public health risk? What are the chances that VRE or the resistance genes will be transmitted from animals to humans? A public health risk must be assumed to exist when transfer from animals to humans can be shown directly or indirectly.

VRE are frequently present in food produced in Denmark as well as in food imported into Denmark from other European countries (23,24). Thus, exposure to humans from insufficiently heated food or cross-contaminated ready-to-eat food takes place. Unlike studies in the United States (21,25), European studies reported that humans frequently are fecal carriers of VRE (26-29). This suggests that VRE can be ingested from food in Europe. Furthermore, VRE was not detected in strict vegetarians in The Netherlands, supporting the view that the source of VRE is contaminated meat (Table 2) (28).

Table 2. Prevalence of vancomycin-resistant *Enterococcus faecium* in fecal samples of residents in a vegetarian and a nonvegetarian nursing home, The Netherlands (28)

| | No. persons investigated | No. *E. faecium* positive | No. VRE positive[a,b] |
|---|---|---|---|
| Vegetarians | 42 | 23 | 0 |
| Nonvegetarians | 62 | 32 | 6 |

[a]P<0.05.
[b]All VRE-positive samples were *E. faecium*.

Molecular typing shows a very high diversity of VRE types in animals as well as humans (30). Nevertheless, similar or related types have been shown to occur in animals and humans on a number of occasions, supporting the assumption that transfer of VRE between humans and animals does take place (18,19). We have recently compared 84 isolates of *E. faecium* from swine, chickens, and humans in Denmark by *Sma*I generated macrorestriction profiles and *Eco*RI ribotyping. Similarity analysis by unweighted pair group method with arithmetic averages–derived dendrograms did not indicate a higher degree of similarity among *E. faecium* isolates (VRE as well as non-VRE) from humans than from animals. This finding indirectly supports the hypothesis that *E. faecium* from different food animals and humans are not discrete populations but belong to a common pool of *E. faecium* shared by animals and humans (data not shown).

The *VanA* gene cluster encoding for vancomycin resistance in animal and human VRE is located on a transposon designated Tn*1546* (31,32). Tn*1546* can easily spread from one enterococcal species to another as well as from enterococci to *S. aureus* (33,34). Recent investigations have documented that in vivo transfer of Tn*1546* can take place in the mammalian intestinal tract (A. Sundsfjord, pers. comm.). Furthermore, animal VRE can colonize the human intestinal tract for at least 3 weeks after experimental ingestion of $10^7$ CFUs of a single strain (35). This indicates that vancomycin resistance can spread in the gastrointestinal tract from transiently colonizing animal VRE to *E. faecium* strains of the resident human gut flora.

The *VanA* gene cluster consists of several genes. We investigated the genes and the regions between them by sequencing of selected areas, polymerase chain reaction amplification of other areas, and hybridization with specific probes (36,37). Thirteen different types were observed. Most differences arose from the presence of insertions or deletions in noncoding intergenic regions. One nucleotide difference was observed in the coding sequences; this point mutation occurred in the *vanX* gene at position 8234, where a G in the reference VRE strain was substituted for a T in some isolates.

In human VRE isolates, this mutation was evenly distributed, whereas in poultry isolates from different countries only the G variant occurred; in isolates from swine from different countries the T variant occurred in nearly all isolates (Table 3). Although we have no explanation for the uneven distribution of subtypes between different animals, the finding of both types in humans does support the hypothesis that animals are a primary source of vancomycin resistance genes in humans, whereas humans apparently do not serve as reservoir for animals, in which case both types would be expected to occur in both animal species. In the same investigation, we found that all human isolates from a Muslim country belonged to the poultry subtype (37). The absence of pork variant types in a Muslim country suggests that food of animal origin is a major reservoir for VRE in humans.

Table 3. Variations in Tn*1546*-like elements of vancomycin-resistant *Enterococcus faecium* isolates of animal and human origin (37)

| Source | No. isolates | T variant | G variant |
|---|---|---|---|
| Humans | 45 | 16 | 29 |
| Pigs | 33 | 32 | 1 |
| Poultry | 193 | 0 | 193 |
| Total | 271 | 48 | 223 |

### The European/American Paradox

Even though the greater frequency of VRE infections in U.S. than in European hospitals would seem to contradict it (12), the hypothesis that animals could serve as reservoirs of human VRE infections is supported by several lines of indirect evidence.

Heavy use of vancomycin (and probably also third-generation cephalosporins) is a prerequisite for frequent VRE infections in hospitals. Heavy use of vancomycin and third generation cephalosporins is more frequent in U.S. than in European hospitals (12,18). Thus, the problem in Europe, irrespective of a high carrier rate of VRE in the community, has not grown to the same proportions as in the United States. VRE infections in the United States are probably due to the heavy use of antibiotics in hospitals and the eventual spread of VRE within and among hospitals by carrier personnel (38).

Another prerequisite for high incidence of VRE hospital infections is a source of VRE. In the United States, primary sources of VRE to hospitals include travelers returning from abroad, tourists, and imported food. Once inside the hospital, VRE can cause nosocomial outbreaks because of its high potential to colonize and persist in the environment, which facilitates its persistence and spread (4). An alternative hypothesis could be that some clones of VRE have a higher potential to cause infections and that such clones are more prevalent in United States. This hypothesis, however, is contradicted by the high number of different types of VRE causing infections in Europe and the United States, which suggests that pathogenic potential is not limited to a few clones of VRE (38).

Avoparcin was approved for growth promotion in Europe in 1974. The first VRE was detected in a human patient in France in 1986. Does this suggest that VRE was not present in animals before 1986? Historically, resistance to growth promoters in animal bacteria was not monitored, and with few exceptions the studies conducted have looked at bacteria other than enterococci because enterococci were not considered foodborne pathogens. Thus, if VRE were not detected in food animals, it may be because they were not looked for.

### The Effect of Prohibiting Use of Avoparcin as a Growth Promoter

Because of public health concerns about resistance to glycopeptide antibiotic drugs, avoparcin was banned in Denmark in 1995. In 1996, Germany took a similar step, and finally in 1997, avoparcin was banned in all EU member states. After the ban in Denmark, a marked reduction in the occurrence of VRE in Danish poultry flocks has been observed at slaughter (from 82% in 1995 to 12% of flocks in 1998; $x^2 = 68.3$ on 5 df.; $p < 0.0001$), whereas in swine only a minor reduction has been observed (Figure) (39). In Germany, a decrease in the incidence of VRE in poultry meat and in fecal samples from humans in the community was observed after discontinuation of avoparcin use in animal husbandry (40). In poultry meat the proportion of VRE-positive samples were reduced from 100% in 1994 to 25% in 1997, and in fecal samples from humans in the community, the carrier rate decreased from 12% in 1994 to 3% in 1997.

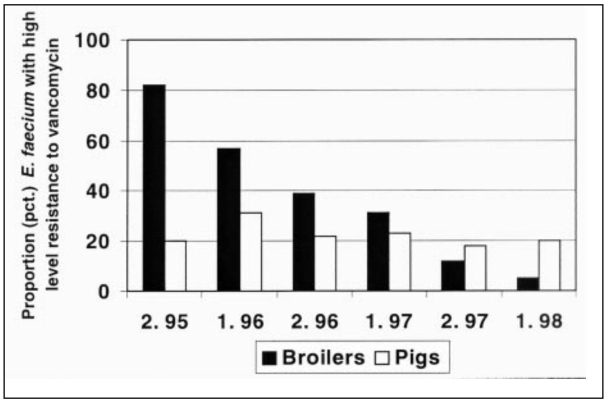

Figure. Trend in the proportion of *Enterococcus faecium* isolates resistant to vancomycin (VRE) during successive half-year periods from second half of 1995 to first half of 1998 (39).

### Similar Problems Related to Other Antimicrobial Growth Promoters

Most of the different growth promoters approved in the EU are active against gram-positive bacteria. With increasing resistance in gram-positive pathogenic bacteria, antimicrobial drugs used as growth promoters have attracted renewed attention as potentially useful for human therapy. More than 10 years after the first VRE was discovered, the first drug for humans with good clinical effect against VRE infections is ready to be marketed. This drug is a combination of two streptogramins, quinupristin and dalfopristin (Synercid).

For decades, virginiamycin, which belongs to the group of streptogramins, has been used as a growth promoter in the European Union as well as in the United States, primarily for poultry production. Investigations in the United States, The Netherlands, and Denmark have frequently found Synercid-resistant *E. faecium* in poultry (41-43). As for VRE, we have no data on the prevalence of streptogramin-resistant *E. faecium* in animal husbandry before virginiamycin was used as a growth promoter. No monitoring has been carried out. Moreover, the gene (*satA*) conferring resistance to virginiamycin and Synercid have been found in animals and humans (44), and in vivo transfer of these genes from resistant to sensitive strains of *E. faecium* in the mammalian gastrointestinal tract has been shown (45). Thus, the events associated with avoparcin and vancomycin may be recurring for Synercid and virginiamycin. Furthermore, the drug anticipated to be next in line after Synercid, a compound called Ziracin, belonging to the class of everninomicins, is practically identical to another growth promoter called avilamycin, which has primarily been used in poultry.

We have detected avilamycin resistance in 69% of *E. faecium* isolates from poultry in Denmark (43). Moreover, preliminary investigations show that resistance to avilamycin gives cross-resistance to Ziracin and that a transferable genetic element may be involved (46). Thus, again use of an antimicrobial drug as a growth promoter may have created a major animal reservoir of resistant *E. faecium,* threatening to shorten the life span of a new promising drug when it is put to use in humans.

### Future Perspectives

At the core of the VRE issue appears to be the way antimicrobial drugs are being developed. New classes of antimicrobial drugs are not available. Instead, old drugs are being modified that may have been used in agriculture as growth promoters for decades because they were not considered useful for humans. Now that physicians are searching for more options in antibiotic treatment, the older drugs may no longer be viable. The use of antimicrobial drugs and development of resistance in animals and humans are interrelated. Therefore, systems to monitor antimicrobial resistance in pathogenic and commensal bacteria should be established. Such systems should cover relevant bacteria from the entire farm-to-fork chain and monitor resistance towards antimicrobial drugs used in both animals and humans, including growth promoters (47-49).

Finally, antimicrobial agents should not be used for growth promotion if they are used in human therapeutics or are known to select for cross-resistance to antimicrobial drugs used in human medicine (47). Antimicrobial agents are too valuable to be used as a tool in animal production because any antimicrobial drug may be useful for human therapy in the future even if not used therapeutically today. Adherence to the World Health Organization recommendations (47) will ensure a systematic approach toward replacing antimicrobial growth promoters with safer nonantimicrobial drug alternatives. The EU countries entered this process in December 1998 when four growth promoters (tylosin, spiramycin, bacitracin, and virginiamycin) were banned because of their structural relatedness to therapeutic antimicrobial drugs used for humans (50).

Dr. Wegener is head of The Danish Zoonosis Centre. His research interests are veterinary public health, microbiology, epidemiology, molecular biology; control of zoonoses, particularly the so-called "modern" bacterial zoonoses; and potential public health consequences of the use of antimicrobial drugs in animal husbandry.

### References

1. Edmond MB, Ober JF, Weinbaum DL, Pfaller MA, Hwang T, Sanford MD, et al. Vancomycin-resistant *Enterococcus faecium* bacteremia: risk factors for infection. Clin Infect Dis 1995;20:1126-33.
2. Leclerq R, Derlot E, Duval J, Courvalin P. Plasmid mediated resistance to vancomycin and teicoplanin in *Enterococccus faecium*. N Engl J Med 1988;319:157-61.
3. Centers for Disease Control and Prevention. Nosocomial enterococci resistant to vancomycin—United States 1989-1993. MMWR Morb Mortal Wkly Rep 1993;42:597-600.
4. Centers for Disease Control and Prevention. Recommendations for preventing the spread of vancomycin resistance. Recommendations of the Hospital Infection Control Practices Advisory Committee (HICPAC). MMWR Morb Mortal Wkly Rep 1995;44:RR12.
5. House of Lords Select Committee on Science and Technology: Resistance to antibiotics and other antimicrobial agents. 1998. London: The Stationary Office; 1998.
6. Bates J, Jordens J, Griffith DT. Farm animals as a putative reservoir for vancomycin resistant enterococcal infections in man. J Antimicrob Chemother 1994;34:507-16.
7. Klare I, Heier H, Claus H, Reissbrodt R, Witte W. VanA-mediated high-level glycopeptide resistance in *Enterococcus faecium* from animal husbandry. FEMS Microbiol Lett 1995;125:165-72.
8. Jensen BB. The impact of feed additives on the microbial ecology of young pigs. Journal of Animal and Feed Sciences 1998;7:45-64.
9. McDonald CL, Kuehnert MJ, Tenover FC, Jarvis WR. Vancomycin-resistant enterococci outside the health care setting: prevalence, sources, and public health. Emerg Infect Dis 1997;3:311-7.
10. Wegener HC. Historical usage of glycopeptides for animals and humans—the American/European paradox revisited. Antimicrob Agents Chemother 1998;42:3049.
11. Witte W. Medical consequences of antibiotic use in agriculture. Science 1998;279:996-7.
12. Kirst HA, Thompson DG, Nicas TI. Historical yearly usage of vancomycin [letter]. Antimicrob Agents Chemother 1998;42:1303-4.
13. Aarestrup FM. Occurrence of glycopeptide resistance among *Enterococcus faecium* isolates from ecological and conventional poultry farms. Microb Drug Resist 1995;1:255-7.
14. Bager F, Madsen M, Christensen J, Aarestrup FM. Avoparcin used as a growth promoter is associated with the occurrence of vancomycin-resistant *Enterococcus faecium* on Danish poultry and pig farms. Prev Vet Med 1997;31:95-112.
15. Devriese LA, Ieven M, Goosens H, Vandamme P, Pot B, Hommez J, et al. Presence of vancomycin-resistant enterococci in farm and pet animals. Antimicrob Agents Chemother 1996;40:2285-7.
16. Tast E, Myllys V, Honkanen-Buzalski T. A survey of resistance to some antimicrobials of enterococcal and *E. coli* strains isolated from pigs and broilers in Finland. In: Proceedings of NKVet Symposium on Antibiotic Resistance. 1997 Nov 7-8. Danish Veterinary Association, Sundvolden, Norway. p. 44.
17. Boisivon A, Vauchel JC, Cheron M, Gobert A, Leturdu F, Chambreuil G, et al. Vancomycin resistant enterococci (VRE) from food animal sources in France. In: Proceedings of the 97th General Meeting of the American Society of Microbiology; 1997 May 4-8; Miami Beach, Florida. Washington: American Society of Microbiology; 1997.
18. van den Bogaard AE, Jensen LB, Stobberingh EE. Vancomycin-resistant enterococci in turkeys and farmers. N Engl J Med 1997a;337:1558-9.
19. Simonsen GS, Haaheim H, Kruse H, Dahl KH, Olsvik Ø, Sundsfjord A. Glycopeptide resistant Enterococci (GRE) at avoparcin-using farms: possible transmission of strains and the *van*A gene cluster between chicken and humans. In: Proceedings of NKVet Symposium on Antibiotic Resistance. 1997 Nov 7-8. Danish Veterinary Association, Sundvolden, Norway. p. 41.
20. Quednau M, Ahrné S, Molin G. Antibiotic resistant enterococci in Swedish and Danish pork and poultry. In: Proceedings of Symposium on Food Associated Pathogens; 1996 May 6-8; The Swedish University of Agricultural Sciences, The Swedish National Committee of Food Science and Technology, and the International Union of Food Science and Technology, Uppsala, Sweden. p. 254.
21. Coque TM, Tomayko JF, Ricke SC, Okhuysen PC, Murray B. Vancomycin-resistant enterococci from nosocomial, community and animal sources in the United States. Antimicrob Agents Chemother 1996;40:2605-9.
22. Thal LA, Chow JW, Mahayni R, Bonilla H, Donabedian SA, Silverman J, Taber S, Zervos MJ. Characterization of antimicrobial resistance in enterococci of animal origin. Antimicrob Agents Chemother 1996;39:2112-5.
23. Wegener HC, Madsen M, Nielsen N, Aarestrup FM. Isolation of vancomycin resistant *Enterococcus faecium* from food. Int J Food Microbiol 1997;35:57-66.
24. Danish Zoonosis Centre. Consumption of antimicrobial agents and occurrence of antimicrobial resistance in bacteria from food animals, food and humans in Denmark. No. 1, Feb 1997. Copenhagen, Denmark: Danish Integrated Antimicrobial Resistance Monitoring and Research Programme (DANMAP).
25. Silverman J, Thal LA, Perri MB, Bostic G, Zervos MJ. Epidemiological evaluation of antimicrobial resistance in community-acquired enterococci. J Clin Microbiol 1998;36:830-2.
26. Van der Auwera P, Pensart N, Korten V, Murray B. Influence of oral glycopeptides on the faecal flora of human volunteers: selection of highly glycopeptide resistant enterococci. J Infect Dis 1996;173:1129-36.

27. Gordts B, Van Landuyt H, Ieven M, Vandamme P, Goossens H. Vancomycin-resistant enterococci colonizing the intestinal tract of hospitalized patients. J Clin Microbiol 1995;33:2842-6.
28. Schouten MA, Voss A, Hoogkamp-Korstanje JAA. VRE and meat. Lancet 1997;349:1258.
29. Ieven M, Vercauteren E, Descheemaeker P, Goosens H. Significant increase in detection of intestinal carriers of glycopeptide resistant enterococci by enrichment cultures [abstract] In: Abstracts of the 37th Interscience Conference on Antimicrobial Agents and Chemotherapy; September 30, 1997 ; Toronto, Canada. Abstract D-118.
30. Van den Brak N, van Belkum A, van Keulen M, Vliegendhart J, Verbrugh HA, Endtz HP. Molecular characterisation of vancomycin-resistant enterococci from hospitalised patients and poultry products in the Netherlands. J Clin Microbiol 1998;36:1927-32.
31. Arthur M, Courvalin P. Genetics and mechanisms of glycopetide resistance in enterococci. Antimcrob Agents Chemother 1993;37:95-112.
32. Aarestrup FM, Ahrens P, Madsen M, Pallesen LV, Poulsen RL, Westh H. Glycopeptide susceptibility among Danish *Enterococcus faecium* and *Enterococcus faecalis* isolates of animal and human origin and PCR identification of genes within the VanA cluster. Antimicrob Agents Chemother 1996;40:1938-40.
33. Leclercq R, Derlot E, Weber M, Duval J, Courvalin P. Transferable vancomycin and teicoplanin resistance in *Enterococcus faecium*. Antimicrob Agents Chemother 1989;33:10-5.
34. Noble WC, Virani Z, Cree RG. Co-transfer of vancomycin and other resistance genes from *Enterococcus faecalis* NCTC 12201 to *Staphylococcus aureus*. FEMS Microbiol Lett 1992;72:195-8.
35. Berchieri A. Intestinal colonization of a human subject by vancomycin-resistant *Enterococcus faecium*. Clin Micrbiol Infect 1999;5:97-100.
36. Jensen LB, Ahrens P, Dons L, Jones RN, Hammerum A, Aarestrup FM. Molecular analysis of the Tn*1546* from vancomycin resistant enterococci isolated from animals and humans. J Clin Microbiol 1998;36:437-42.
37. Jensen LB. Differences in the occurrence of two base pair variants of Tn*1546* from vancomycin-resistant enterococci from humans, pigs and poultry. Antimicrob Agents Chemother 1998;42:2463-4.
38. Thal L, Donabedian S, Robinson-Dunn B, Chow JW, Dembry L, Clewell DB, et al. Molecular analysis of glycopeptide-resistant *Enterococcus faecium* isolates collected from Michigan hospitals over a 6-year period. J Clin Microbiol 1998;36:3303-8.
39. Bager F, Aarestrup FM, Madsen M, Wegener HC. Glycopeptide resistance in *Enterococcus faecium* from broilers following discontinued use of avoparcin. Microb Drug Resist 1999;5:(in press).
40. Klare I, Badstübner D, Konstabel C, Böhme G, Claus H, Witte W. Decreased incidence of VanA-type vancomycin-resistant enterococci isolated from poultry meat and from fecal samples of humans in the community after discontinuation of avoparcin usage in animal husbandry. Microb Drug Resist 1999;5 (in press).
41. Welton LA, Thal LA, Perri MB, Donabedien S, McMahon J, Chow JW, et al. Antimicrobial resistance in enterococci isolated from turkey flocks fed virginiamycin. Antimicrob Agents Chemother 1998;42:705-8.
42. Van den Bogaard AE, Mertens P, London NH, Stobberingh EE. High prevalence of vancomycin- and pristinamycin-resistant enterococci in healthy humans and animals in The Netherlands: is the addition of antibiotics to animal feed to blame? Antimicrob Agents Chemother 1997;40:454-6.
43. Aarestrup FM, Bager F, Madsen M, Jensen NE, Meyling A, Wegener HC. Surveillance of antimicrobial resistance in bacteria isolated from food animals to growth promoters and related therapeutic agents in Denmark. APMIS 1998;106:606-22.
44. Hammerum AH, Jensen LB, Aarestrup FM. Detection of the SatA gene and transferability of virginiamycin resistance in *Enterococcus faecium* from food animals. FEMS Microbiol Lett 1998;168:145-51.
45. Jakobsen BM, Skou M, Hammerum AM, Jensen LB. In vivo transfer of the satA gene between isogenic strains of *Enterococcus faecium* in the mammalian gastrointestinal tract. In: Proceedings of the Second World Congress on Anaerobic Bacteria and Infections; 1998 Oct 3-6; Nice, France.
46. Aarestrup FM. Association between decreased susceptibility to a new antibiotic for treatment of human diseases; everninomicin (SCH 27899), and resistance to an antibiotic used for growth promotion in animals, avilamycin. Microb Drug Resist 1998;4:137-41.
47. The medical impact of the use of antimicrobials in food animals. Report from a WHO meeting; Berlin, Germany 1997 Oct 13-17. Geneva: World Health Organization; 1997.
48. The role of international trade in animals, animal products and feed in the spread of transferable antimicrobial resistance and possible methods for control of the spread of infectious agent resistance factors. In: Proceedings of the 18th Conference of the Office International des Epizooties (OIE) Regional Commission of Europe; 1998 Sep 22-25; Prague, Czech Republic.
49. The Copenhagen recommendation. Report from the invitational EU conference on the microbial threat; 1998 Sep 9-10; Copenhagen, Denmark. (Internet address http://www.sum.dk/publika/micro98/index.htm).
50. Commission regulation of amending council directive 70/524/EEC concerning additives in feedingstuffs as regards withdrawal of the authorisation of certain antibiotics. Document No.: VI/7767/98. European Commission, Brussels, Belgium.

## References

[PDF_00622] Aarestrup F. M., Ahrens P., Madsen M., Pallesen L. V., Poulsen R. L., Westh H. (1996). Glycopeptide susceptibility among Danish Enterococcus faecium and Enterococcus faecalis isolates of animal and human origin and PCR identification of genes within the VanA cluster.. Antimicrob Agents Chemother.

[PDF_00534] Bager F., Madsen M., Christensen J., Aarestrup F. M. (1997). Avoparcin used as a growth promoter is associated with the occurrence of vancomycin-resistant Enterococcus faecium on Danish poultry and pig farms.. Prev Vet Med.

[PDF_00497] Centers for Disease Control and Prevention (CDC) (1993). Nosocomial enterococci resistant to vancomycin--United States, 1989-1993.. MMWR Morb Mortal Wkly Rep.

[PDF_00574] Coque T. M., Tomayko J. F., Ricke S. C., Okhyusen P. C., Murray B. E. (1996). Vancomycin-resistant enterococci from nosocomial, community, and animal sources in the United States.. Antimicrob Agents Chemother.

[PDF_00490] Edmond M. B., Ober J. F., Weinbaum D. L., Pfaller M. A., Hwang T., Sanford M. D., Wenzel R. P. (1995). Vancomycin-resistant Enterococcus faecium bacteremia: risk factors for infection.. Clin Infect Dis.

[PDF_00494] Leclercq R., Derlot E., Duval J., Courvalin P. (1988). Plasmid-mediated resistance to vancomycin and teicoplanin in Enterococcus faecium.. N Engl J Med.

[PDF_00628] Leclercq R., Derlot E., Weber M., Duval J., Courvalin P. (1989). Transferable vancomycin and teicoplanin resistance in Enterococcus faecium.. Antimicrob Agents Chemother.

[PDF_00632] Noble W. C., Virani Z., Cree R. G. (1992). Co-transfer of vancomycin and other resistance genes from Enterococcus faecalis NCTC 12201 to Staphylococcus aureus.. FEMS Microbiol Lett.

[PDF_00606] Schouten M. A., Voss A., Hoogkamp-Korstanje J. A. (1997). VRE and meat.. Lancet.

[PDF_00592] Silverman J., Thal L. A., Perri M. B., Bostic G., Zervos M. J. (1998). Epidemiologic evaluation of antimicrobial resistance in community-acquired enterococci.. J Clin Microbiol.

[PDF_00579] Thal L. A., Chow J. W., Mahayni R., Bonilla H., Perri M. B., Donabedian S. A., Silverman J., Taber S., Zervos M. J. (1995). Characterization of antimicrobial resistance in enterococci of animal origin.. Antimicrob Agents Chemother.

[PDF_00647] Thal L., Donabedian S., Robinson-Dunn B., Chow J. W., Dembry L., Clewell D. B., Alshab D., Zervos M. J. (1998). Molecular analysis of glycopeptide-resistant Enterococcus faecium isolates collected from Michigan hospitals over a 6-year period.. J Clin Microbiol.

[PDF_00596] Van der Auwera P., Pensart N., Korten V., Murray B. E., Leclercq R. (1996). Influence of oral glycopeptides on the fecal flora of human volunteers: selection of highly glycopeptide-resistant enterococci.. J Infect Dis.

[PDF_00583] Wegener H. C., Madsen M., Nielsen N., Aarestrup F. M. (1997). Isolation of vancomycin resistant Enterococcus faecium from food.. Int J Food Microbiol.

[PDF_00556] van den Bogaard A. E., Jensen L. B., Stobberingh E. E. (1997). Vancomycin-resistant enterococci in turkeys and farmers.. N Engl J Med.

[PDF_00614] van den Braak N., van Belkum A., van Keulen M., Vliegenthart J., Verbrugh H. A., Endtz H. P. (1998). Molecular characterization of vancomycin-resistant enterococci from hospitalized patients and poultry products in The Netherlands.. J Clin Microbiol.

