# Erratum Vol. 5 No. 3

**DOI:** 10.3201/eid0506.990627

**Published:** 1999


### 4th Decennial International Conference on Nosocomial and Health Care-Associated Infections
**Atlanta, Georgia, USA, March 5–9, 2000**

### On-Line Abstract Submission Deadline—November 15, 1999

Every 10 years, the Centers for Disease Control and Prevention sponsors the International Conference on Nosocomial and Health Care-Associated Infections in Atlanta, Georgia. The 1970, 1980, and 1990 conferences set the agenda for research and infection control practices for the respective decades; the Fourth Decennial Conference will set the agenda for the new millennium.

### Call for abstracts

The deadline for on-line submission of abstracts is November 15, 1999. The deadline for abstracts submitted by mail is November 1, 1999. Selected late-breaker abstracts must be submitted by Monday, January 17, 2000. Abstracts can be submitted on-line starting in June at http://www.decennial.org/. Abstract submission forms can be downloaded from the Internet site or mailed upon request. Abstract submission forms will be automatically distributed to all Association for Professionals in Infection Control and Epidemiology (APIC), Infectious Disease Society of America (IDSA), and Society for Health Care Epidemiology of America (SHEA) members, as well as attendees at the Interscience Conference on Antimicrobial Agents and Chemotherapy (ICAAC).

### Program Registration

Program and registration information will be distributed to all members of APIC, IDSA, and SHEA, as well as to ICAAC attendees. If you are not a member of one of these organizations, please contact the 4th Decennial Conference (see below) and ask to be placed on the mailing list. The conference will be held in conjunction with the 10th annual meeting of SHEA; separate registration is required for SHEA events.

For more information contact the 4th Decennial Conference, 6220 Montrose Road, Rockville, MD 20852, USA; telephone: 301-984-9450; fax: 301-984-9441; e-mail: info@decennial.org; Internet: www.decennial.org.

### Towards a Healthy Europe for the Year 2010
**V European Conference on Health Promotion and Health Education**
**Santander, Spain, May 10-13, 2000**

The theme of cultural diversity has been chosen for this conference, which will deal with professional practices, communication in social settings, and health policies that should be developed to achieve a healthy Europe for the next millennium.

We believe that the coexistence of different cultures is sometimes the cause of health problems but also increasingly the source of solutions and opportunities for health promotion and for the achievement of a healthy Europe.

The subjects related to this central theme will be addressed in plenary sessions, open debates, oral presentations, and poster sessions. There will also be workshops which will serve as a forum for professionals with common interests in specific areas.

For additional information, please contact Asociación de Educación para la Salud (ADEPS), Servicio de Medicina Preventiva, 4ª Norte, Hospital Clínico San Carlos, c/o Profesor Martín Lagos, 28040 Madrid, Spain; telephone: 34-91-330-3422; fax: 34-91-543-7504; e-mail: msainz@hcsc.es.

**Erratum Vol. 5 No. 3**

In the article "Use of Antimicrobial Growth Promoters in Food Animals and *Enterococcus faecium* Resistance to Therapeutic Antimicrobial Drugs in Europe, " by H.C. Wegener et al., reference 3 is incorrect. The correct reference is

3. Centers for Disease Control and Prevention. Summary of Notifiable Diseases, United States, 1997. MMWR Morb Mortal Wkly Rep 1998; 46:1-87.

We regret any confusion this error may have caused.

In the article "Use of Antimicrobial Growth Promoters in Food Animals and *Enterococcus faecium* Resistance to Therapeutic Antimicrobial Drugs in Europe, " by H.C. Wegener et al., reference 3 is incorrect. The correct reference is

3. Centers for Disease Control and Prevention. Summary of Notifiable Diseases, United States, 1997. MMWR Morb Mortal Wkly Rep 1998; 46:1-87.

We regret any confusion this error may have caused.

